# Phlebotomy resulting in controlled hypovolemia to prevent blood loss in major hepatic resections (PRICE-2): study protocol for a phase 3 randomized controlled trial

**DOI:** 10.1186/s13063-022-07008-y

**Published:** 2023-01-18

**Authors:** Guillaume Martel, Tori Lenet, Christopher Wherrett, François-Martin Carrier, Leah Monette, Aklile Workneh, Karine Brousseau, Monique Ruel, Michaël Chassé, Yves Collin, Franck Vandenbroucke-Menu, Élodie Hamel-Perreault, Michel-Antoine Perreault, Jeieung Park, Shirley Lim, Véronique Maltais, Philemon Leung, Richard W. D. Gilbert, Maja Segedi, Jad Abou-Khalil, Kimberly A. Bertens, Fady K. Balaa, Tim Ramsay, Dean A. Fergusson

**Affiliations:** 1 Liver and Pancreas Unit, Department of Surgery, The Ottawa Hospital– General Campus, University of Ottawa, 501 Smyth Road, CCW 1667, Ottawa, ON K1H 8L6 Canada; 2 Clinical Epidemiology Program, Ottawa Hospital Research Institute, Ottawa, ON Canada; 3 Departments of Anesthesiology and Pain Medicine, The Ottawa Hospital, University of Ottawa, Ottawa, ON Canada; 4 Department of Anesthesiology, Centre Hospitalier de l’Université de Montréal, Université de Montréal, Montréal, QC Canada; 5 Department of Medicine, Critical Care Division, Centre Hospitalier de l’Université de Montréal, Université de Montréal, Montréal, QC Canada; 6 Division of General Surgery, Department of Surgery, Centre Hospitalier Universitaire de Sherbrooke, Sherbrooke, QC Canada; 7 Hepato-Pancreato-Biliary and Liver Transplantation Surgery Unit, Department of Surgery - Centre Hospitalier de l’Université de Montréal, Montréal, QC Canada; 8 Departement of Anesthesiology, Centre Hospitalier Universitaire de Sherbrooke, Sherbrooke, QC Canada; 9 Department of Anesthesiology and Perioperative Care, Vancouver General Hospital, University of British Columbia, Vancouver, BC Canada; 10 Department of Surgery, Vancouver General Hospital, University of British Columbia, Vancouver, BC Canada

**Keywords:** Hepatectomy, Liver resection, Phlebotomy, Hypovolemia, Hemodilution, Blood transfusion, Blood loss, Autologous blood transfusion, Randomized controlled trial

## Abstract

**Introduction:**

Blood loss and red blood cell (RBC) transfusion in liver surgery are areas of concern for surgeons, anesthesiologists, and patients alike. While various methods are employed to reduce surgical blood loss, the evidence base surrounding each intervention is limited. Hypovolemic phlebotomy, the removal of whole blood from the patient without volume replacement during liver transection, has been strongly associated with decreased bleeding and RBC transfusion in observational studies. This trial aims to investigate whether hypovolemic phlebotomy is superior to usual care in reducing RBC transfusions in liver resection.

**Methods:**

This study is a double-blind multicenter randomized controlled trial. Adult patients undergoing major hepatic resections for any indication will be randomly allocated in a 1:1 ratio to either hypovolemic phlebotomy and usual care or usual care alone. Exclusion criteria will be minor resections, preoperative hemoglobin <100g/L, renal insufficiency, and other contraindication to hypovolemic phlebotomy. The primary outcome will be the proportion of patients receiving at least one allogeneic RBC transfusion unit within 30 days of the onset of surgery. Secondary outcomes will include transfusion of other allogeneic blood products, blood loss, morbidity, mortality, and intraoperative physiologic parameters. The surgical team will be blinded to the intervention. Randomization will occur on the morning of surgery. The sample size will comprise 440 patients. Enrolment will occur at four Canadian academic liver surgery centers over a 4-year period. Ethics approval will be obtained at participating sites before enrolment.

**Discussion:**

The results of this randomized control trial will provide high-quality evidence regarding the use of hypovolemic phlebotomy in major liver resection and its effects on RBC transfusion. If proven to be effective, this intervention could become standard of care in liver operations internationally and become incorporated within perioperative patient blood management programs.

**Trial registration:**

ClinicalTrials.gov NCT03651154. Registered on August 29 2018.

**Supplementary Information:**

The online version contains supplementary material available at 10.1186/s13063-022-07008-y.

## Background

Major liver resections are increasingly used to treat both malignant and benign conditions [1]. They constitute a highly complex procedure owing to their technical difficulty and significant perioperative morbidity. Despite improvements in postoperative mortality, the overall incidence of perioperative complication rates remains as high as 50% in major series [2–5]. Although patients undergoing liver surgery have benefited from tremendous improvements in resection techniques, anesthesia, and critical care, these operations remain fraught with complications, attributable in part to the risk of major intraoperative blood loss and hemorrhage.

Major blood loss and the associated risk of red blood cell (RBC) transfusion remain significant concerns for surgeons, anesthesiologists, and patients [6, 7]. Among high-volume institutions, patients having a liver resection carry a risk of RBC transfusion of 17–41% [2–5, 8, 9]. Though a life-saving intervention when used appropriately, RBC transfusion can also be associated with infectious disease transmission, life-threatening transfusion reactions, and delayed recovery and worsened long-term cancer-specific survival [10–17]. A reduction in the use of RBC transfusions has thus been identified as an important priority for liver surgeons [18, 19], as well as by various influential interest groups such as Choosing Wisely, Choosing Wisely Canada, and the Canadian Blood Services [20–23].

Several cardiovascular, pharmacologic, behavioral, and surgical interventions can minimize blood loss in liver surgery, and consequently RBC transfusions [7, 24, 25]. Unfortunately, the evidence base supporting each intervention is highly variable. In clinical practice, surgeons and anesthesiologists utilize a widely variable combination of these techniques, based on personal experience and interpretation of the literature. Liver resection performed under conditions of low central venous pressure (CVP) (<5 cm H_2_O) is widely regarded as the standard of care [26–30]. Canadian liver surgeons have reported targeting a low CVP frequently or always during liver resection [18]. A low CVP is thought to be associated with a low proximal pressure in the hepatic veins, through a low pressure in the right atrium and inferior vena cava. This may lead to less pronounced blood loss from hepatic vein branches during the liver parenchymal transection, when the largest proportion of blood loss in liver surgery occurs and is most likely to be influenced by the volume status of the patient and associated CVP [6]. Intraoperative CVP can be controlled by the anesthesiologist using a variety of techniques. Most commonly, the patient is kept relatively intravascularly volume depleted by minimizing intravenous (IV) fluid administration. Other techniques can be used to supplement the use of IV fluid management, such as hypoventilation, as well as the use of diuretics or venous vasodilating agents [31, 32].

Hypovolemic phlebotomy (HP) is a relatively novel and underreported intervention that can be used to decrease blood loss and blood transfusion in liver surgery. It consists of the removal of whole blood from the patient prior to the initiation of liver transection, without replacement by IV fluid. Following resection, the phlebotomized blood is re-transfused to the patient. It is proposed that HP leads to a decrease in the net circulating blood volume and portal blood flow resulting in a mild to moderate decrease in CVP [33]. Acute phlebotomy of 8 mL/kg decreases the circulating blood volume by 10–12% [34], a state that is comparable to donating blood or to class 1 hemorrhagic shock [35].

A preliminary review of the literature reveals a paucity of in data on HP. A systematic review examining all cardiovascular interventions to decrease blood loss and blood transfusion in liver resection was published and last updated in January 2012 [24]; it identified 10 trials comprising 617 patients and examined a variety of techniques including low CVP, autologous blood donation, acute normovolemic hemodilution, and hypoventilation. The authors concluded that further randomized clinical trials with low risks of bias are required. Similarly, a recent network meta-analysis assessing all methods used to reduce blood loss in liver resections identified 67 randomized controlled trials examining methods such as low CVP and surgical techniques [36]. The results were inconclusive, and HP was noticeably absent from the data.

Recent surveys of liver surgeons and existing systematic reviews suggest that HP is not widely used, likely because its effectiveness has not been demonstrated [18, 37]. Our group initially acquired observational experience with 45 patients who underwent HP prior to elective major liver resection [38, 39]. In this cohort, HP of 5.3–10.1 mL/kg was associated with decreased blood loss compared to 101 patients without HP (5.9 vs. 6.7 mL/kg, *p*=0.042) and was strongly associated with decreased transfusion on multivariable analysis (odds ratio [OR] = 0.23, 95% confidence interval [CI] 0.08 to 0.66, *p*=0.006). Our feasibility trial, PRICE-1, further demonstrated the possibility of effectively implementing HP in the context of a clinical trial, with 62 patients successfully randomized. Although not powered to detect a difference in clinical outcomes, the ensuing results signaled a lower incidence of RBC transfusion among patients at high risk of transfusion (13% vs. 33%) [40]. Results from other observational studies have suggested that HP may lower CVP in major liver resection and decrease blood loss and transfusion risk [41, 42]. Finally, in a survey of worldwide liver surgery centers, a small number of centers reported using HP when hepatic vein bleeding was excessive during liver transection [37]. Small studies in liver transplantation, living donor hepatectomy, and more recently with our own data, have suggested that HP has the potential to significantly reduce blood transfusion in elective liver surgery [39, 41, 43, 44]. Finally, the safety profile of this intervention appears excellent [40, 42, 45].

While preliminary observations and cohort studies suggest a benefit from HP in liver surgery, it is unclear whether the observed reduction in RBC transfusion is truly attributable to this intervention [46–48]. It is also unknown whether any observed reduction in RBC transfusion will translate into improvement in other perioperative outcomes such as the ease of surgical resection, postoperative complications, or surgical mortality. Finally, more rigorous acquisition of safety data is required. These considerations highlight the need for a definitive randomized controlled trial.

It is hoped that the current trial, summarized in the following protocol, will provide robust evidence of effectiveness of HP in preventing RBC transfusion in liver surgery. As one of the largest trials ever carried out in this patient population, a superiority result would have an immediate impact on the treatment of patients undergoing liver surgery and influence worldwide practice. On the contrary, if the trial fails to demonstrate effectiveness, then this intervention can be abandoned, as the question will have been answered definitively.

### Research question

In patients undergoing major liver resection, what is the effect of hypovolemic phlebotomy compared to usual care on 30-day red blood cell transfusion? It is hypothesized that HP is superior to usual care with respect to 30-day RBC transfusion.

## Methods and design

### Study design

PRICE-2 is a double-blind multicenter randomized controlled trial (RCT) to evaluate the superiority of HP over usual care. Patients will be randomly allocated to the intervention (HP + usual care) or to the control (usual care). Enrolment will occur at four Canadian academic liver surgery hospitals. Eligible patients that provide consent will be randomized to either of the two study arms in a 1:1 allocation ratio. Apart from the trial intervention, management of patients prior, during, and after liver surgery will be at the discretion of individual practitioners. The trial has been registered publicly at ClinicalTrials.gov since August 2018 (NCT03651154). The trial protocol will be reported in accordance with the Standard Protocol Items: Recommendations for Interventional Trials (SPIRIT) guidelines (Additional file 1).

### Enrollment

Enrolment started at the coordinating center (The Ottawa Hospital) in October 2018, while enrolment at the second site started in December 2018 (Centre Hospitalier de l’Université de Montréal). Enrolment at the third site started in June 2019 (Centre Hospitalier Universitaire de Sherbrooke). Enrolment at the fourth site started in January 2022 (Vancouver General Hospital). Recruitment is anticipated to be completed in the first or second quarter of 2023.

### Eligibility criteria

Adult patients scheduled to undergo a major liver resection by either laparotomy or laparoscopy for any indication will be eligible. Major liver resection is defined liberally based on the PRICE-1 pilot RCT [40] as resection or partial resection of at least 3 liver segments, right posterior sectionectomy (segments 6 and 7), central resection of segments 4b/5, or a resection of a full single segment in a patient known to have liver cirrhosis.

Exclusion criteria were adopted and modified from Jarnagin et al. [49]. Individuals who meet one or more of the following criteria will be excluded from the study: (1) age <18 years; (2) preoperative hemoglobin <100 g/L; (3) glomerular filtration rate (GFR) <60 mL/min; (4) platelets count <100 × 10^9^/L; 5) active cardiac conditions (defined as unstable coronary syndromes, severe valvular disease, myocardial infarction within the past 6 months, or hypertrophic cardiomyopathy); (6) history of significant cerebrovascular disease (clinically-significant cerebrovascular accident within the past 6 months or severe carotid stenosis >70%); (7) history of significant peripheral vascular disease (non-revascularized with regular/ongoing claudication); (8) pregnancy; (9) refusal of blood product transfusions; (10) presence of active infection; (11) preoperative autologous blood donation; (12) planned intraoperative use of cell salvage; (13) inability to comply with the trial follow-up at 30 and 90 days; and (14) previous participation in the trial in the case of redo liver resections.

### Interventions

#### Experimental intervention

The experimental intervention will consist of usual care plus HP. A CVP ≤ 5 cm H_2_O will be targeted as part of usual care. HP will consist of the withdrawal of 7–10 mL/kg of whole blood from the patient in a blood donation bag, as tolerated (e.g., for a 70-kg patient, 490 to 700 mL of whole blood). A range of volumes is provided to allow leeway for the anesthesiologist in managing the hemodynamic effects of the intervention. The withdrawn volume will be calculated based on the specific gravity of blood [50]. In the case of patients with a body mass index (BMI) greater than 30, the volume of blood to be withdrawn will be calculated based on lean-scaled body weight (LBW) calculated using sex and BMI (see Additional file 2) [51]. LBW will be calculated using a convenient online calculator and outputted as Normalized Lean Weight [52]. Following phlebotomy, the volume of removed blood will not be replaced by the administration of IV fluids; hemodynamic disturbances will be managed using vasopressors [34, 39, 43, 48, 53]. If the patient were to require an unplanned transfusion of RBC or fresh frozen plasma (FFP) during the parenchymal transection portion of the operation, the anesthesiologist will first administer the phlebotomized whole blood followed by allogeneic blood products, at their discretion. The phlebotomized whole blood will always be transfused back after liver transection before the conclusion of the operation regardless of blood loss.

HP will be carried out using standard transfusion medicine precautions for the handling of blood products. Whole blood collection bags will be obtained and appropriately labelled with the patient’s name and hospital identifier. Blood tubing, tubing clamps, and a digital scale will be available in the operating room. HP can be carried out using any vascular access route, at the anesthesiologist’s discretion. Most commonly, blood will be withdrawn from the central venous catheter (CVC), although other options include a 14-gauge antecubital IV (or other large proximal vein/IV caliber), a large-bore central rapid infusion catheter, or an arterial catheter. HP should be initiated and completed prior to the onset of liver parenchymal transection, following trial allocation. Strict aseptic technique will be maintained. The blood tubing system must remain closed and sealed to prevent backflow of air or other potential contaminants. The target blood volume will be determined and translated into weight. This weight of whole blood will then be phlebotomized using a dedicated zeroed scale. If necessary, Trendelenburg position can be used to maximize venous return. Blood collection bags should be frequently and gently agitated to facilitate mixture with the citrate phosphate dextrose adenine (CPDA) anticoagulant. Like other blood products, whole blood bags should be placed in a dedicated transfusion medicine cooler once collected. Collected blood must be re-infused within 8 h if kept in a cooler, or within 4 h if kept at room temperature.

#### Control intervention

The control intervention will consist of usual care, which typically involves the minimization of IV fluid administration. A CVP ≤ 5 cm H_2_O will be targeted as part of usual care. The use of diuretics or vasodilatory agents will not be permitted under the protocol.

#### Combined oncology cases

For patients undergoing combined oncologic operations (e.g., liver and colon), the liver resection portion of the procedure will be conducted first to avoid any potential hemodynamic or regional blood flow issues during the colonic resection. Once the surgeon indicates that the parenchymal transection portion of the liver resection is completed, the anesthesiologist will re-infuse any phlebotomized whole blood back using standard transfusion precautions before colonic resection. All intraoperative outcomes (e.g., blood loss) will be assessed for the entire duration of the anesthetic.

#### Common anesthetic management

In all patients, epidural anesthesia may be used at the discretion of the anesthesiologist. If an epidural is utilized, it will be inserted prior to the induction of general anesthesia and the anesthesiologist will test its functionality at the start of surgery using a test dose of local anesthetics as per local practices. The administration of additional local anesthetic within the epidural will be proscribed until completion of liver transection, after which point the epidural may be used at the anesthesiologist’s discretion. This limitation was introduced to avoid the sympathetic blockage associated with local anesthetic agents administered within epidurals. Tranexamic acid will be administered to all patients unless contraindicated (deep vein thrombosis or pulmonary embolism within the last 12 months). A 1g bolus will be administered at the start of surgery, followed by a 1g infusion administered over the remainder of surgery. A central venous catheter (CVC) will be inserted in all patients. The anesthesiologist should determine the choice of HP vascular access before revealing trial allocation, to prepare all patients for the intervention without the knowledge of the intervention allocation. The use of any other monitoring techniques will be at the anesthesiologist’s discretion (e.g., non-invasive hemodynamic cardiac output monitoring, arterial catheter, cerebral oximetry).

#### Transfusion protocol

A transfusion protocol will be used to ensure that only clinically appropriate RBC transfusions are administered. The Ottawa Criteria for Appropriate Transfusion in Hepatectomy (OCATH) [54] will be used in the operating room, and the AABB Guidelines [55] will be followed in the postoperative setting. At individual study sites, study patients will be identified to all practitioners (including trainees) and the transfusion protocol will be displayed on patient charts.

### Outcomes

#### Primary outcome

The primary outcome will be the proportion of patients receiving at least one unit of allogeneic RBC within 30 days of randomization.

#### Secondary outcomes

Secondary outcomes (Table 1) will also be considered up to 30 days and include:

1. Proportion of patients receiving other allogeneic blood products (FFP, cryoprecipitate, platelets, and albumin) and the number of units of transfused allogeneic blood products.
2. Blood loss, measured in the operating room using the methodology employed in the feasibility trial [40]. All suctioned fluids will be recorded. All unused irrigation fluids will be similarly measured and subtracted from the total irrigation fluid provided for the case. All surgical sponges will be weighed using a dedicated zeroed scale and converted to milliliters from grams using the specific gravity of blood. Total blood loss will thus be measured as: suctioned fluids + sponge volume − irrigation fluids. Blood loss will also be estimated using the preoperative and day 2 hemoglobin values (accounting for any transfused allogeneic blood) using the Flordal equation [56, 59].
3. All intraoperative and postoperative adverse events, graded according to the Clavien-Dindo Classification of Surgical Complications [57] and the Comprehensive Complication Index [58]. In addition, end-organ ischemic complications (renal [60], cardiac, cerebral, hepatic, mesenteric) will be specifically monitored, graded, and reported.
4. Intraoperative physiologic parameters (CVP, pulse pressure variation [PPV], cardiac index, total peripheral vascular resistance index, mean arterial pressure [MAP], urine output). All parameters will be measured at the onset of surgery after induction of anesthesia, and at different time points during the intraoperative period.
5. Mortality at 30 and 90 days.
6. Oncologic outcomes will be examined retrospectively approximately 3 years following completion of the study. A separate study protocol will be devised to examine these outcomes.

**Table 1:** Outcome measures and their evaluation timepoints

Outcome measures	Timepoints of evaluation
**Blood loss**: Three methods will be used independently. In the operating room, all blood and fluid aspirated from the abdomen will be measured accurately using graduated suction containers. The amount of irrigation fluid will be carefully monitored and recorded. The weight of all surgical sponges will be measured. This information will be used by (1) the surgeon and (2) anesthesiologist to independently visually estimate blood loss, as is commonly done in clinical practice. In parallel, intraoperative blood loss will also be (3) calculated based on the Flordal equation [56], using preoperative and day 2 hemoglobin levels.	Day 0 Day 2
Packed RBC transfusion	Day 7 Day 30
Other blood product transfusion rate (FFP, cryoprecipitate, albumin, others)	Day 7 Day 30
Overall morbidity rate, major morbidity rate (Clavien-Dindo grade 3a or greater [57]), Comprehensive Complication Index [58] and any perioperative adverse events.	Day 7 Day 30
Mortality	Day 30
Surgeon perception scale (Additional file 3)	Day 0
Intraoperative physiologic parameters (CVP, PPV, total peripheral vascular resistance index, MAP, urine output)	Measured at the onset of surgery (after induction of anesthesia) at various time points

*CVP* Central venous pressure, *FFP* Fresh frozen plasma, *MAP* Mean arterial pressure, *PPV* Pulse pressure variation, *RBC* Red blood cell

### Participant timeline

The treatment period will be limited to the duration of liver surgery. Patients will be followed during their index admission. Study data will be obtained from the chart and electronic medical record. Following discharge, the patient will be seen in clinic by the surgeon, in accordance with local practice. At 30 and 90 days, the study coordinator (SC) will complete the follow-up by verifying the medical record and contacting the patient by phone. If the patient reports additional outpatient clinic visits, transfusions, or re-admission to hospital, relevant records will be obtained and abstracted. The primary outcome of transfusion is objective and readily identifiable from the electronic medical record and institutional blood bank data.

### Sample size

A review of observational data from The Ottawa Hospital collected between 2010 and 2016 suggests that the cumulative incidence of any perioperative blood transfusion among patients undergoing liver resection is 24% (*n*=316) [41]. Similarly, data from 85 participating NSQIP hospitals in 2013 documented a transfusion incidence of 22% (*n*=2448) [9], whereas a Canadian multicentre collaborative documented 26.5% (*n*=1287) [61]. Our group has documented a transfusion rate of 11.1% in an observational cohort of 45 patients with HP [41]. PRICE-2 is powered to detect a clinically important decrease in perioperative 30-day blood transfusion incidence of 20 to 10% with HP, at 80% power and 5% alpha error (199 patients per arm). A relative risk reduction of 50% has been previously targeted in other transfusion trials in major abdominal surgery [62]. PRICE-2 will include one Haybittle-Peto interim analysis, which does not require sample size adjustment. Allowance is made for a 5% rate of partial protocol compliance or non-compliance in the intervention arm, yielding an 11% increase in total sample size based on the Lachin formula [63]. This trial will thus recruit until 440 patients have been randomized and have had a liver resection after excluding patients that were enrolled and randomized but who did not undergo a liver resection due to disease progression and unresectability identified at surgery (modified intention-to-treat analysis).

### Recruitment procedures

**Fig. 1:**
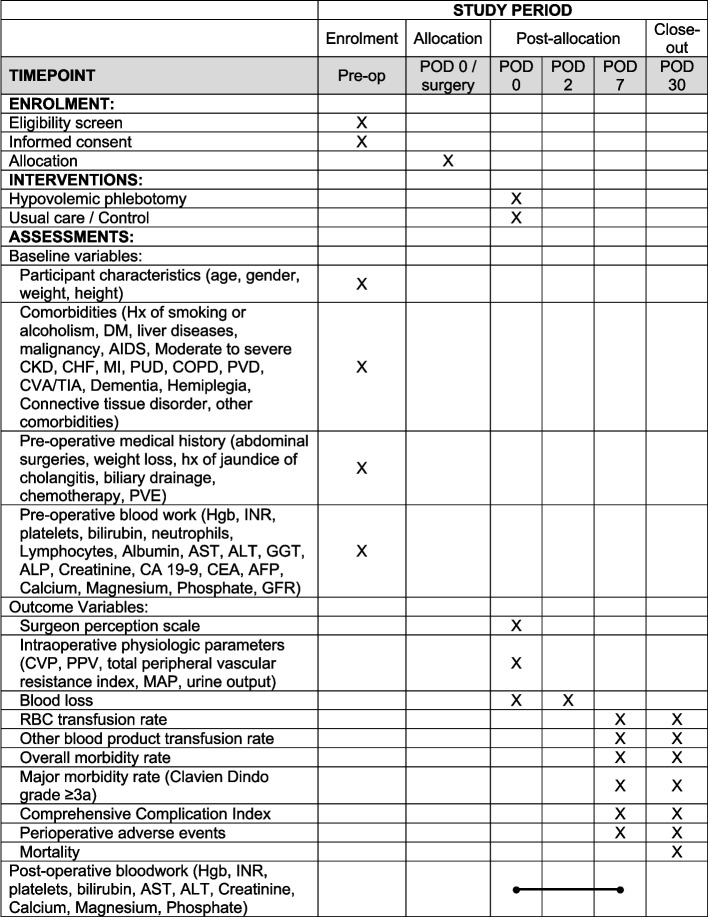
Schedule of enrollment, interventions, and assessments. POD, postoperative day; Hx, history; DM, diabetes mellitus; AIDS, acquired immunodeficiency syndrome; CKD, chronic kidney disease; CHF, congestive heart failure; MI, myocardial infarction; PUD, peptic ulcer disease; COPD, chronic obstructive pulmonary disease; PVD, peripheral vascular disease; CVA, cardiovascular accident; TIA, transient ischemic attack; PVE, portal vein embolization; Hgb, hemoglobin; INR, international normalized ratio; AST, aspartate transaminase; ALT, alanine transaminase; GGT, gamma-glutamyl transpeptidase; ALP, alkaline phosphatase; CA 19-9, carbohydrate antigen 19-9; CEA, carcinoembryonic antigen; AFP, alpha-fetoprotein; GFR, glomerular filtration rate; CVP, central venous pressure; PPV, pulse pressure variation; MAP, mean arterial pressure; RBC, red blood cell

Patients will be considered for enrolment at four tertiary Canadian academic hospitals (The Ottawa Hospital, Centre Hospitalier de l’Université de Montréal, Centre Hospitalier Universitaire de Sherbrooke, and Vancouver General Hospital). All patients considered for elective liver resection will be seen by an attending surgeon in the outpatient clinic and assessed for study eligibility. Once the patient has provided informed consent for surgery, they will be screened for trial eligibility. Written informed consent will be obtained by study coordinators. Informed consent forms will be available in French and English. Figure 1 shows the schedule of enrollment, interventions, and assessments throughout the trial.

### Assignment of interventions

#### Sequence generation

Patients will be randomized in a 1:1 allocation ratio to the two arms of the study, with the use of permuted blocks of variable length (2, 4, and 6). Randomization will be stratified by center. The Ottawa Methods Centre (OMC) will prepare the computer-generated randomization sequence. Secure central web-based randomization will allow for the verification of enrolment and eligibility, as well as ensure allocation concealment.

#### Allocation concealment

The use of central web-based randomization with permutated blocks will ensure that individual surgeons and anesthesiologists cannot determine trial allocation ahead of time. Allocation to the experimental or control interventions will take place the day of surgery. The anesthesiologist will reveal the allocation once the patient is considered “anesthesia ready,” which implies that general anesthesia has been administered, and all lines and catheters have been inserted. By revealing the allocation once the patient is in the operating room and under anesthesia, we can ensure that clinical care will not be influenced by trial allocation.

#### Coordination

The trial will be coordinated out of the Ottawa Hospital Research Institute (OHRI). Each study center will have a site investigator and SC. The Ottawa SC will also act as the overall trial coordinator. Each SC will be responsible for the (a) screening and consenting of patients, (b) central randomization of patients in the operating room, (c) proper implementation of trial procedures, and (d) following patients and correctly filling electronic data collection.

### Blinding

Patients will be blinded to the intervention, as allocation will be revealed to the anesthesiology team following induction of general anesthesia. The anesthesiologist will not be blinded, as they will carry out the intervention. The surgeon, surgical trainees, and the nursing team will be blinded to the intervention. Blinding of the surgical and nursing teams will be completed by carrying out all physical steps necessary for HP (including equipment setup) prior to revealing allocation. Blood collection and scripted movements for the control group (including reverse Trendelenburg position) will take place behind large sterile drapes. At the time of re-infusion of the whole blood, collection bags and tubing will remain hidden from view behind the large drapes. Blinding procedures have been successfully implemented in 98% of patients in the feasibility trial [40]. Success was confirmed based on a post-procedural questionnaire administered to surgeons. Because most perioperative transfusions occur once liver surgery is completed, blinding of the surgeon/surgical team is important to maintain objectivity in determining the need for a blood transfusion. Although the anesthesiologist cannot be blinded, the use of a transfusion protocol specific to the operating room environment will ensure that any transfusion administered is appropriate and clinically indicated [54]. At any time during the study, the attending anesthesiologist can choose to unblind the surgeon for safety reasons. Postoperative outcome assessors will also be blinded to the intervention.

### Data collection methods

#### Assessment and collection of outcomes

Case report forms (CRFs) were created in collaboration with the surgical and anesthesiology teams to capture all relevant study data. The CRFs consist of (1) demographics and medical history details, (2) intraoperative data—surgeon, (3) intraoperative data—anesthesiologist, and (4) postoperative data. These will contain source data. These CRFs are incorporated into an electronic data capture system (EDCS) set up by the OMC. The forms were informed by feasibility trial conducted prior to PRICE-2 [40].

The surgeon, anesthesiologist, and SC will be responsible for the completion of specific sections of the CRFs. The surgeon and anesthesiologist will fill out their respective intraoperative data. The SC will collect demographic data, medical history, and postoperative data.

#### Training

Given that HP is commonly employed at all study sites, no additional training is required, as both surgical and anesthesiology teams will be performing tasks that are within their scope of practice. Training will be provided with respect to study procedures.

#### Intervention and patient compliance

The intervention will be administered in the operating room while the patient is undergoing liver surgery and general anesthesia. Patient compliance is thus not a significant issue to consider. Compliance with the assigned intervention will be monitored carefully. Compliance with the experimental intervention will require that the patient receive HP at a targeted volume of 7–10 mL/kg. *Partial compliance* will be defined as receiving an incomplete (<7 mL/kg) or excessive (>10 mL/kg) phlebotomy. *Non-compliance* will be defined as not receiving the allocated trial intervention (crossover). This can include patients randomized to the experimental intervention who do not receive HP, or patients randomized to the control intervention who receive HP due to perceived excessive blood loss during surgery. In the feasibility trial, there was no case of non-compliance [40]. Partial compliance is expected to be a rare occurrence, as all study sites have significant experience with using HP in clinical practice. In the feasibility trial, partial compliance with HP occurred in less than 5% of cases. Modifications were made to the technical aspects of the protocol to simplify whole blood collection. The PRICE-2 sample is adjusted to reflect partial compliance in 5% of patients.

Cell salvage can sometimes be used to avoid allogeneic RBC transfusions in usual practice [64]. Cases of liver resection where cell salvage is intended to be used are contraindicated in this trial. It is possible that surgeons may elect to use cell salvage in the case of major intraoperative hemorrhage. Surgeons are advised not to do so under the protocol and no such case occurred in the pilot trial. In the event of unplanned cell salvage use, any blood that is retrieved from the operative field and transfused will be coded as a perioperative RBC transfusion event towards the primary outcome, as it is reasoned that the unintended use of emergency cell salvage was likely utilized by the team due to major blood loss to avoid excessive allogeneic transfusions. On the contrary, blood that is salvaged but not transfused will not be considered as a transfusion event.

#### Loss to follow-up

Losses to follow-up to 30 days are not expected following liver surgery. All patients are carefully monitored in hospital for an average of 5–7 days, followed by 1–2 prescribed clinic visits within 3 weeks of discharge.

### Data management

Patients will be identified by a participant ID number on all study documents and electronic databases. All sites will keep any hard copy study documents in locked and secure drawers at their respective sites. For each study patient, relevant preoperative, intraoperative, and postoperative data will be collected and entered into CRFs within the EDCS. Data quality and accuracy will be routinely checked by the trial coordinator.

### Statistical analysis

Our analyses will estimate a modified intention-to-treat effect, such that patients who were randomized in the operating room but did not undergo liver resection due to unresectability will not be included in the analyses. A blinded committee of liver surgeons will adjudicate each post-randomization exclusion. This will ensure that only cases of liver resection abandoned due to unresectability will be excluded from analysis. All other patients who have had protocol deviations (e.g., having an unplanned minor rather than major resection) or crossover (e.g., inability to achieve a full 7–10 mL/kg phlebotomy) will be analyzed within their allocated arm based on an intention-to-treat principle.

Continuous variables will be reported using means and standard deviations, or medians and interquartile ranges, as appropriate. Categorical variables will be reported as frequencies and percentages.

For the primary outcome of 30-day cumulative incidence of any perioperative RBC transfusion, the risk ratio and risk difference will be presented with their respective 95% confidence intervals. Both unadjusted and adjusted effect estimates will be presented. We will fit multivariable generalized linear models using generalized estimating equations (GEE) with an exchangeable correlation matrix with model-based (non-robust) standard errors to estimate an adjusted risk ratio and relative risk, accounting for center effect (clusters). We will incorporate important prognostic covariates (age, sex, preoperative anemia, indication for surgery, magnitude of liver resection, extra-hepatic procedures). Although safeguards are in place to ensure complete data acquisition throughout the trial, missing data for covariates will be handled using multiple imputations by chained equations, where multiple imputed datasets will be pooled to generate final estimates using Rubin’s rules. We will also conduct a complete case analysis to evaluate the potential effect of the missing at random assumptions underlying missing data. Hypothesis testing for the primary endpoint will be carried out at a two-tailed alpha level of 0.05.

For secondary outcomes, both unadjusted and adjusted measures of effect will be presented. Risk ratios and mean differences with 95% confidence intervals will be presented for dichotomous and continuous data, respectively. We will use similar models with GEEs and model-based (non-robust) standard errors to generate adjusted effect estimates, incorporating the same set of covariates as for the primary outcome. For secondary outcomes and analyses, no adjustment will be made for multiplicity.

Subgroup analyses will be conducted based on known risk factors for transfusion in liver surgery: preoperative anemia (larger treatment effect expected with anemia), indication for surgery (larger treatment effect expected with primary liver cancers), and magnitude of liver resection (larger treatment effect expected for extended resections) [61]. An interaction test will be used between each subgroup variable and the treatment group within GEEs estimating risk differences. Published criteria will be used to evaluate the plausibility of the subgroup effects [65].

### Data monitoring

The PRICE-2 Steering Committee will be comprised of the principal investigator (PI), all co-investigators (Co-I), and the trial coordinator. The Steering Committee will communicate regularly and will meet as needed. The Committee will implement the trial, review recruitment milestones, and manage any evolving concerns related to trial procedures. An independent DSMB will be appointed and will have expertise in surgery, anesthesiology, biostatistics, and clinical trials. The roles, responsibilities, and reporting of the DSMB, including the interpretation of the interim analysis and adverse events, will be adapted from the DAMOCLES Charter [66].

Three interim analyses will be completed and reported to the Data Safety Monitoring Board (DSMB). The first interim analysis will take place after 100 patients and will examine accrual and adverse events data. The second interim analysis will be conducted after 200 patients and will examine accrual, adverse events, and the primary efficacy outcome. A conservative Haybittle-Peto boundary (*p*<0.001) [67] will be utilized. The third interim analysis will be conducted after 300 patients and will examine accrual and adverse events. The final analysis will be conducted once all 440 patients have been randomized, have had a liver resection, and have been followed to 30 days following surgery, based on the modified intention-to-treat principle.

Study site monitoring will take place remotely at the start of recruitment for each site, as well as upon completion of accrual. Ongoing data monitoring will be conducted and data queries that arise, as well as any issues related to incomplete data, will be addressed on an ongoing basis with study sites. Finally, a formal trial audit plan has been created. A formal audit of the trial will be conducted by the study sponsor and will include complete review of the coordinating center study file, review of a small proportion of participant records, and review of the study files of participating sites.

### Adverse events

Published data from our group and others have not documented any significant differences in overall postoperative complications, end-organ ischemic complications (renal, cardiac, cerebral, hepatic, and mesenteric), or perioperative mortality between HP and usual care [34, 39, 41–45, 48]. Similarly, PRICE-1 [40] and two other small trials [46, 47] noted no significant differences in any adverse event metric. Nevertheless, low-volume phlebotomy could potentially lead to any physiologic effect associated with blood loss. These effects could have implications for any major organ system due to decreased perfusion (e.g., myocardial ischemia, cerebrovascular accident, acute renal failure, hepatic insufficiency). These risks exist with liver surgery, whether HP is performed or not, due to potential significant blood loss. To be considered fit for liver surgery, patients cannot have excessive comorbid illnesses that could be grossly exacerbated by the operation. The patient population that is thus considered for surgery is a relatively fit one that can tolerate HP. Furthermore, the inclusion and exclusion criteria that have been built in this protocol exclude those at a greater risk of adverse event associated with decreased organ perfusion. Other potential risks associated with HP pertain to the collection of whole blood and its auto-transfusion at the end of surgery. There is a potential risk for clerical error with the blood. Similarly, there is a theoretical risk of bacterial contamination of the collection bag, tubing, and, whole blood. To minimize these risks, all study procedures have been developed conjointly between surgery, anesthesiology, and the Transfusion Medicine (TM) department at our center and have been agreed upon by participating centers. The collection bags will be provided by TM, labelled with the patient identifier, and handled and processed in the same manner as any other blood product in the operating room. This procedure will allow for the safe handling of all blood specimens, in accordance with all protocols already in place at each study center

The PI or co-investigators will determine if any serious and unexpected adverse events occur from randomization to postoperative day 30. The SC, PI, or co-investigators will examine changes in laboratory values, vital signs, and clinical data, and will determine if the changes are clinically important and different from what is expected during treatment of participants having undergone hepatic surgery and general anesthesia. As perioperative complications represent an important secondary outcome in this trial, adverse events (AEs) will be carefully monitored and recorded. AEs can generally be described as any unfavorable and unintended sign, symptom, or disease temporarily associated with the treatment, whether or not related to the study treatment.

For this trial, any surgical, medical, or anesthesia-related AE that deviates from the usual postoperative course of patients undergoing major liver resection will be collected and recorded in the study AE Source Form by the PI, Co-I, or SC. The PI or Co-I will then assign a grading to this AE based on the Clavien-Dindo classification of perioperative complications [57]. The following will not be considered AEs:

- *Intraoperative*: bleeding (unless deemed unusually severe by the surgeon), hypotension (unless deemed unresponsive to usual mitigation strategies), oliguria
- *Postoperative*: pain, hypotension (unless deemed unresponsive to usual mitigation strategies), oliguria (unless associated with acute kidney injury), nausea or vomiting (unless related to ileus or obstruction, or if refractory), sore throat, muscle aches, pruritus, chills, chest pain (unless deemed to be related to a defined AE such as acute coronary syndrome), anemia not requiring transfusion, electrolyte disturbances (unless refractory to usual replacement therapy)

Any AE that is graded as Clavien-Dindo grade 3a or greater will be considered a severe adverse event (SAE). SAEs will be recorded on the study SAE Case Report Form. All SAEs will be further assessed by the PI or Co-Is for expectedness and relatedness to the study procedures. On that basis, unexpected SAEs will be reported to the REB.

### Dissemination and protocol amendments

Any protocol amendment will be submitted to the relevant REBs for approval before implementation and registered at ClinicalTrials.gov. Once REB approval is obtained at the coordinating center, the PI will brief all relevant parties’ site investigators and coordinators on the amendments, and a copy of the amended protocol will be provided. Participating sites will then seek approval of the amendment from their respective hospital’s REB before implementing the updated protocol. Study results will be disseminated using peer-reviewed publications.

## Discussion

### Perspectives of the study

PRICE-2 is a complex interventional trial conducted in the operative environment. There are several practical challenges associated with carrying out trials of this nature, such as “defining, developing, documenting, and reproducing complex interventions” [68]. Each challenge has been carefully thought out and addressed in the study protocol. First, this study requires a significant degree of cooperation among team members (surgeons, anesthesiologists, nurses, blood bankers, clinicians in charge of PBM programs). Second, components of the intervention have to be sufficiently defined to allow reproducibility but must remain pragmatic enough to allow for patient management in real time in the operating room. Third, numerous co-interventions can influence the primary outcome of RBC transfusion, as well as the associated secondary outcome of blood loss. In the interest of striking a judicious balance, the investigators have chosen to focus on the most important co-interventions, namely administering tranexamic acid to all patients, disallowing other CVP-lowering techniques (e.g., diuretics, vasodilators, local anesthetic within epidurals), and disallowing cell salvage use. Fourth, the investigators seek to include all patients at high risk of bleeding by introducing a liberal definition of major hepatic resection. Finally, learning curve considerations have led the investigators to limit opening this trial within centers with previous HP experience.

The results of this randomized control trial will provide high-quality evidence regarding the use of hypovolemic phlebotomy in major liver resection and its effects on RBC transfusion. If proven to be effective, this intervention could become standard of care in liver operations internationally and become incorporated within perioperative patient blood management programs.

### Update on trial progress and challenges

Trial enrolment was initiated in October 2018. Total accrual has been expected to last 4 years. At present accrual rate, PRICE-2 is expected to be closed to enrolment on the second quarter of 2023. Accrual has slowed down at the onset of the Covid-19 pandemic (spring 2020) and then again in mid 2021, owing to operating room access restrictions at some of the study sites.

## Trial status

Protocol version 3.3, dated June 7, 2022

Date recruitment began: October 2018

Estimated date of recruitment completion: second quarter of 2023

## Supplementary Information

**Additional file 1.** SPIRIT reporting checklist for protocol of a clinical trial.

**Additional file 2.** Lean-scaled body weight (LBW) [51].

**Additional file 3.** Surgeon Perception Scale.

## Data Availability

The datasets analyzed during the current study and statistical code are available from the corresponding author on reasonable request, as is the full protocol.

## Abbreviations

AE: Adverse event
BMI: Body mass index
CI: Confidence interval
CIHR: Canadian Institutes of Health Research
Co-I: Co-investigator
CPDA: Citrate phosphate dextrose adenine
CRF: Case report forms
CVC: Central venous catheter
CVP: Low central venous pressure
DSMB: Data Safety Monitoring Board
EDCS: Electronic data capture system
FFP: Fresh frozen plasma
GFR: Glomerular filtration rate
HP: Hypovolemic phlebotomy
ICMJE: International Committee of Medical Journal Editors
IV: Intravenous
LBW: Lean-scaled body weight
MAP: Mean arterial pressure
MICE: Multivariate imputation by chained equations
NSQIP: National Surgical Quality Improvement Program
OCATH: Ottawa Criteria for Appropriate Transfusions in Hepatectomy
OHRI: Ottawa Hospital Research Institute
OMC: Ottawa Methods Center
OR: Odds ratio
PI: Principal investigator
PPV: Pulse pressure variation
RBC: Red blood cell
RCT: Randomized controlled trial
REB: Research ethics board
SAE: Severe adverse event
SC: Study coordinator
SPIRIT: Standard Protocol Items: Recommendations for Interventional Trials
TM: Transfusion medicine
