# Supplementary material for: Phlebotomy resulting in controlled hypovolemia to prevent blood loss in major hepatic resections (PRICE-2): study protocol for a phase 3 randomized controlled trial
Source: Trials. 2023 Jan 18;24:38. doi: 10.1186/s13063-022-07008-y (PMC9848035; doi:10.1186/s13063-022-07008-y)
Supplement: Supplementary file 2 — Additional file 2. Lean-scaled body weight (LBW) [51]. [file 13063_2022_7008_MOESM2_ESM.docx]

**Additional File 2:** Lean-scaled body weight (LBW) [51]

| LBW = real weight x LSF |
| --- |

Whereby LSF is the lean-scaled factor; with

| LSF_female_ =______14,148_______  8,780 + (244 x BMI) |
| --- |

| LSF_male_ =______11,432_______  6,680 + (216 x BMI) |
| --- |

BMI: body mass index; LBW: lean-scaled body weight; LSF: lean-scaled factor
