# Supplementary material for: Phlebotomy resulting in controlled hypovolemia to prevent blood loss in major hepatic resections (PRICE-2): study protocol for a phase 3 randomized controlled trial
Source: Trials. 2023 Jan 18;24:38. doi: 10.1186/s13063-022-07008-y (PMC9848035; doi:10.1186/s13063-022-07008-y)
Supplement: Supplementary file 3 — Additional file 3. Surgeon Perception Scale. [file 13063_2022_7008_MOESM3_ESM.docx]

**Additional File 3:** Surgeon Perception Scale

**To be filled by the surgeon immediately following liver parenchymal transection**

Please rate the overall ease/difficulty with which this liver resection was completed:

1 2 3 4 5 6 7 8 9 10

Easiest Average Hardest

Please rate the ease/difficulty with which the liver parenchymal transection was completed:

1 2 3 4 5 6 7 8 9 10

Easiest Average Hardest

Please rate your impression of blood loss during the parenchymal transection:

1 2 3 4 5 6 7 8 9 10

None/minimal Average Most/excessive

Please rate your impression of the filling status of the hepatic veins during the parenchymal transection:

1 2 3 4 5 6 7 8 9 10

Empty/flat Average Full/excessive

**Surgeon signature: ___________________________________________________**

**Date: _______________________________________________________________**
